# Cognitive bias modification for social anxiety: a living systematic review and network meta-analysis

**DOI:** 10.12688/wellcomeopenres.25609.2

**Published:** 2026-05-25

**Authors:** Jaycee Kennett, Virginia Chiocchia, Claire Friedrich, Simon E. Blackwell, Per Carlbring, Toshi Furukawa, Spyridon Siafis, Catherine Money, Thomy Tonia, Simonne Wright, Kelvin Opiepie, Richardson Mojica, Olufisayo Elugbadeo, Paulina Schenk, Micaela Santilli, Susan Michie, Janna Hastings, Hossein Dehdarirad, Claire Stansfield, James Thomas, Jennifer Potts, Georgia Salanti, Andrea Cipriani

**Affiliations:** 1 Department of Psychiatry, University of Oxford, Oxford, UK; 2 Oxford Precision Psychiatry Lab, NIHR Oxford Biomedical Research Centre, Oxford, England, UK; 3 EPPI Centre, Social Research Institute, University College London, London, England, UK; 4 Institute of Social and Preventive Medicine, University of Bern, Bern, Switzerland; 5 Department of Clinical Psychology and Experimental Psychopathology, Institute of Psychology, University of Göttingen, Göttingen, Germany; 6 Department of Psychology, Stockholm University, Stockholm, Sweden; 7 Department of Health Promotion and Human Behaviour, Kyoto University School of Public Health Department of Health Informatics, Kyoto, Japan; 8 Department of Psychiatry and Psychotherapy, TUM School of Medicine and Health, Technical University of Munich, Munich, Germany; 9 German Center for Mental Health, München/Augsburg, Germany; 10 Department of Psychiatry, Stellenbosch University, Stellenbosch, Western Cape, South Africa; 11 Global Lived Experience Advisory Board, GALENOS, Ogrun State, Nigeria; 12 Global Lived Experience Advisory Board, GALENOS, Bacoor, Philippines; 13 South African Medical Council Unit on the Genomics of Brain Disorders, Department of Psychiatry, Stellenbosch University Faculty of Medicine and Health Sciences, Cape Town, South Africa; 14 Department of Psychiatry, University of Ibadan College of Medicine, Ibadan, Nigeria; 15 Centre for Behaviour Change, University College London, London, UK; 16 Institute for Implementation Science in Health Care, Faculty of Medicine, University of Zurich, Zurich, Zurich, Switzerland; 17 School of Medicine, University of St. Gallen, St. Gallen, Switzerland; 18 Swiss Institute of Bioinformatics, Lausanne, Switzerland; 19 NIHR Oxford Health Clinical Research Facility, Oxford Health NHS Foundation Trust, Warneford Hospital, Oxford, UK

**Keywords:** Cognitive bias modification, social anxiety, CBM, attentional bias, interpretation bias

## Abstract

**Background:**

Cognitive Bias Modification (CBM) is a potentially effective intervention for social anxiety disorder (SAD). However, the relative efficacy of CBM subtypes remains uncertain.

**Methods:**

We co-produced a living systematic review of controlled studies evaluating stand alone CBM in participants with SAD and in non-clinical participants undergoing a social stressor, seeking to triangulate these. Databases were searched until October 2025 and two independent reviewers screened, extracted and critically appraised studies. The primary outcome was reduction in SAD symptoms, but we collected data on other mental health outcomes. Random-effect network meta-analysis was conducted. Credibility was assessed using the Confidence In Network Meta-Analysis framework.

**Results:**

CBM for attention (CBM-A) demonstrated the largest reduction in SAD symptoms relative to control CBM-A although this estimate was imprecise (Standardised Mean Difference (SMD) = −0.18, 95% CI: −0.41 to 0.05, 95% PI: −1.02 to 1.04). Opposite CBM-A, training attention towards negative stimuli, performed similarly (SMD -0.17, 95% CI: −0.65 to 0.31, 95% PI: −0.97 to 1.21). CBM for interpretation (CBM-I) reduced SAD symptoms (SMD = −0.47, 95% CI: −0.84 to −0.09, 95% PI: −0.53 to 1.46) and cognitive bias versus its task-matched control condition without active bias training. The SAD network showed high inconsistency and 95% of studies were at moderate or high risk of bias. Three comparisons involving CBM-A were rated as moderate confidence, all others were low or very low. Sparse data precluded NMA of outcomes in non-SAD participants.

**Conclusion:**

Inconsistency and imprecision in the network preclude firm conclusions regarding CBM efficacy for SAD. CBM-A showed a small effect but its similarity to opposite CBM-A suggests benefits may not depend on training direction. Future studies should explore the mechanism of CBM and reporting on potential side-effects. We will update this living systematic review as part of the GALENOS project.

**Registration:** PROSPERO-ID:CRD42024601380.

## Plain Language summary

Social anxiety is a condition involving fears of being judged, criticised, or embarrassed. Cognitive Bias Modification (CBM) is computerised training that aims to change unhelpful thinking patterns that may contribute to poor mental health. Some CBM programmes train people to shift attention away from negative information, while others focus on encouraging positive or neutral interpretations of social situations. However, it is unclear whether certain subtypes of CBM work better than others. We examined whether CBM reduces symptoms of social anxiety and whether different subtypes vary in effectiveness, by collecting and reviewing evidence published up to October 2025.

For individuals with social anxiety, attention-based CBM emerged as one of the more effective subtypes, but outcomes did not differ depending on whether attention was directed toward positive, neutral, or negative information. This raises questions about how attention-based CBM works as we expected the direction of the training to determine this type of CBM’s effectiveness. Interpretation-based CBM showed some efficacy, but the reduction in social anxiety symptoms was small. We assessed our confidence in most of our results to be low or very low due to the limited evidence available.

Few eligible studies involved individuals without social anxiety, limiting conclusions about CBM as a preventative or early intervention. No CBM subtype showed efficacy over any other for other mental-health outcomes. We also found data on side effects was poorly reported.

Overall, we cannot make any firm conclusions about the effectiveness of CBM, however the results show the importance of understanding the mechanism behind attention-based CBM. To confidently assess how effective CBM is, more evidence is needed, particularly how social anxiety is changed and whether the training itself has side-effects. We will update this review regularly as part of the GALENOS project.

## Background

Anxiety is characterised by excessive worry, anticipatory apprehension, and heightened physiological arousal that is often disproportionate to an individual’s situation ( American Psychiatric Association, 2022; WHO, 2022). When anxiety arises specifically in contexts that involve actual or perceived social evaluation (such as having a conversation or performing in front of others), it can be understood as social anxiety ( WHO, 2022). The distress associated with social anxiety may be linked to fears of negative judgement, embarrassment, or humiliation and exceeds the objective threat posed by the environment, the situation, and sociocultural expectations ( Morrison & Heimberg, 2013). Should symptoms of social anxiety be persistent and impair an individual’s quality of life or functioning, it can be diagnosed as social anxiety disorder (SAD) ( American Psychiatric Association, 2022). Individuals with SAD have been shown to preferentially attend to negative information about social information (known as attentional bias), to interpret ambiguous information about social situations more negatively (known as interpretation bias), and to remember experiences involving social situations more negatively (memory bias) ( Stopa, 2009). These cognitive biases have been thought to play a causal role in the development and maintenance of SAD, as they can create a self-reinforcing cycle of sensitivity to negative social information and promoting social withdrawal ( Hofmann, 2007).

Cognitive Bias Modification (CBM) refers to a class of interventions that aim to systematically alter attention, interpretation, and memory biases via simple repetitive training procedures such as selecting the location of a flashing dot on a screen and in doing so, may improve symptoms of mental disorders ( Vrijsen *et al.*, 2024). Within the umbrella term of CBM there are numerous subtypes which intend to alter different biases. Using CBM-A (Cognitive Bias Modification for Attention), some studies have shown to reduce symptoms of SAD ( Amir *et al.*, 2009; Julian *et al.*, 2012; Schmidt *et al.*, 2009); however other studies have failed to replicate these findings ( Liu *et al.*, 2017). CBM-I (Cognitive Bias Modification for Interpretations) has similarly shown mixed findings ( Fodor *et al.*, 2020). Nevertheless, there is evidence that CBM interventions may be effective in reducing symptoms of a number of mental disorders that involve biased information processing such as depression and addiction ( Boffo *et al.*, 2019; Fodor *et al.*, 2020).

Subtypes of CBM aim to train different cognitive biases and using varying procedures; as such, the relative efficacy of these contrasting subtypes is insufficiently understood. Mostly delivered by computer, if effective CBM represents a potentially cost-effective way to deliver treatment for SAD, particularly in areas where systemic, financial, and infrastructural barriers constrain access to other treatments such as cognitive behavioural therapy. As CBM has the potential to be scalable and requires low resources to deliver, evaluating the efficacy of this class of interventions is crucial. Another advantage of CBM, is that it is made up of specific ‘ingredients’ which can be substituted or altered and therefore CBM may lend itself more to straightforward iterative refinement ( Holmes *et al.*, 2018). By contrast, other treatments for SAD such as psychological therapies (considered the gold standard in SAD treatment) are complex and individualised interventions, drawing on numerous psychological paradigms, making them more difficult to refine and improve in a systematic way ( Pelissolo *et al.*, 2019).

A better understanding of the literature on CBM interventions in the context of SAD is key to improving and refining these interventions. Also crucial is disentangling both the relative efficacy of different subtypes of CBM and identifying the potential mechanisms of these subtypes. Thereby, better guidance can be provided on which CBM subtypes are more likely to be effective and how they should be tailored to work through specific mechanisms.

This living systematic review (LSR) will use MacLeod and Mathews (2012) definition of cognitive bias, defining it as “a systematic selectivity in information processing that operates to favour one type of information over another” (p2). To examine the association between CBM and symptoms of SAD, we drew on two sources of evidence; a) individuals with diagnosed SAD (hereafter referred to as the clinical source of evidence) and b) individuals not recruited for having a mental health condition (hereafter referred to as the non-clinical source of evidence). The non-clinical source of evidence included people who may have elevated levels of social anxiety/sub-clinical social anxiety but who have not been diagnosed with SAD. While CBM is often used to augment other interventions, we have chosen to include stand-alone CBM only in order to isolate the relative efficacy of these interventions for social anxiety. This review took an intentionally narrow approach and as the review progresses into living mode and is updated, we may decide to include trials in which CBM augments other psychological interventions.

## Research questions

1. What are the effects of CBM on reducing SAD symptom severity in individuals with SAD?
2. What are the effects of CBM on changing SAD related symptoms in individuals not diagnosed with any mental health condition?
3. What mediates the effects of CBM on SAD on both individuals with SAD and individuals not diagnosed with a mental health condition?

## Methods

### Eligibility criteria

We included randomised controlled trials investigating the effects of CBM versus an inactive or active control in participants with either a) SAD (as measured by clinical diagnosis through any operationalised criteria or above-threshold symptoms on any standardised rating scale) or b) no mental or physical health diagnoses. We defined CBM interventions as interventions that target any cognitive bias, directly altering a specific cognitive bias by repeatedly exposing individuals to tasks that promote predetermined patterns of selective processing ( MacLeod & Mathews, 2012). The population, intervention, comparator, and outcomes that were included for both sources of evidence are detailed in Table 1 and Table 2.

**Table 1.** Study inclusion and exclusion criteria for the clinical source of evidence: individuals with Social anxiety disorder.

*Study design*	*We included:* • Randomised controlled trials • Studies published in any language and in any year
*Population*	*We included:* • Participants with a clinical diagnosis of social anxiety disorder or above-threshold symptoms on any standardised scale used to diagnose social anxiety disorder • Participants of any age *We excluded:* • Participants with social anxiety disorder as a secondary diagnosis and who were recruited based on another mental health diagnosis • Participants who are undergoing any other psychological treatment during the study period
*Experimental* *interventions/exposures*	*We included:* • An intervention of CBM, structured and designed to alter a cognitive bias related to social situations. We will accept any number of sessions or any delivery format • Interventions which include multi-component CBM * We excluded:* • CBM as augmentation of a pharmacological or psychological intervention which occur concurrently
*Control interventions/ non-exposures *	*We included:* • Inactive controls such as a) waitlist control or b) no intervention • Non-specific controls such as a) sham CBM, b) placebo training, c) attention control condition, d) neutral control condition (i.e. a task that does not involve any CBM), or e) other interventions that aim to control for aspects of the CBM intervention such as time spent completing tasks or interaction with the experimental setup, but which are not intended to affect cognitive biases • Negative controls (where participants are trained towards the opposite cognitive bias to the intervention) *We excluded:* • Any form of current psychotherapy (defined as a directed psychological intervention designed to improve distress)
*Outcomes*	Studies will be included irrespective of outcomes reported. *Primary outcome:* • Change in SAD severity from pre to post intervention *Secondary outcomes:* • Acceptability (number of drop-outs due to any reason) • Tolerability (number of drop-outs due to adverse events) • Number of participants reporting any adverse events • Number of participants reporting each specific adverse event. To mitigate variability in how adverse events are reported we will use the Medical Dictionary for Regulatory Activities to harmonise and organise terminology related to adverse events • Change in cognitive bias measured as self-report measures or attentional or interpretive bias tasks • Clinical global impression of improvement • Change in diagnostic status (no longer meeting diagnostic criteria) • Change in general anxiety levels ( *e.g.* GAD-7, BAI) • Change in general depression levels ( *e.g.* PHQ-9, BDI-II) • Change in quality of life/measures of subjective wellbeing • Change in symptoms of social functioning

CBM = Cognitive Bias Modification; SAD = Social Anxiety Disorder; GAD-7 = Generalised Anxiety Disorder-7; BAI = Beck Anxiety Inventory; PHQ-9 = Patient Health Questionnaire-9; BDI-II = Beck Depression Inventory-2.

**Table 2.** Study inclusion and exclusion criteria for the non-clinical source of evidence: individuals without any mental health condition.

* Study design*	*We included:* • Randomised controlled trials • Studies published in any language and in any year
*Population*	*We included:* • Participants who have not been recruited based on having a mental health diagnosis • Participants of any age • Participants exposed to an analogue social stressor during the study (i.e. a task or situation that aims to induce mild social anxiety) *We excluded:* • Participants who are undergoing any other psychological treatment during the study period
*Experimental interventions/ exposures*	*We included:* • An intervention of CBM, structured and designed to alter a cognitive bias relating to stressful social situations. We will accept any number of sessions or any delivery format • Interventions which include multi-component CBM *We excluded:* • CBM as augmentation of a pharmacological or psychological intervention which occurs concurrently
*Control interventions/non- exposures*	*We included:* • Inactive controls such as waitlist control or no intervention • Non-specific controls such as a) sham CBM, b) placebo training, c) attention control condition, d) neutral control condition (i.e. a task that does not involve any CBM) or e) other interventions that aim to control for aspects of the CBM intervention such as time spent completing tasks or interaction with the experimental setup, but which are not intended to affect cognitive biases • Negative controls (where participants are trained towards the opposite cognitive bias to the intervention) *We excluded:* • Any form of current psychotherapy (defined as a directed psychological intervention designed to improve distress)
*Outcomes*	Studies were included irrespective of outcomes reported. *Primary outcome:* • Change in social anxiety-related symptoms, defined below *Secondary outcomes:* • Acceptability (number of drop-outs due to any reason) • Tolerability (number of drop-outs due to adverse events) • Number of adverse events • Number of participants with a specific adverse event to mitigate variability in how adverse events are reported we will use the Medical Dictionary for Regulatory Activities to harmonise and organise terminology related to adverse events • Change in cognitive bias

CBM = Cognitive Bias Modification.

### Outcomes

Our primary outcome of interest was the change in SAD symptom severity from pre- to post-intervention, measured with validated clinician-rated or self-reported measures. Our secondary outcomes were change in symptoms of depression, symptoms of general anxiety (all measured as above), change in bias measured by attentional or interpretive tasks or with self-reported measures, and quality of life, and social functioning. We also sought data on dropouts due to any reason (acceptability), dropouts due to adverse events (tolerability), and details of any adverse events reported.

### Study identification

The search strategy was defined in collaboration with the search team and informed by prior research on CBM interventions ( Fodor *et al.*, 2020; Martinelli *et al.*, 2022). The primary approach in developing the search strategy focussed on searching titles, abstracts, keywords and database-controlled vocabulary on the concepts of CBM and studies with a randomised experimental design. To enhance sensitivity, a range of terms for CBM were used.

We searched the following databases covering health, psychology and multi-disciplinary research fields: CENTRAL (Cochrane Library), Embase (OVID), MEDLINE (OVID), PsycINFO (OVID), Scopus, Web of Science (SCI, SSCI, ESCI and the related conference and book indexes). LILACS, WHO Index Medicus, the trial registries ( ClinicalTrials.gov, ScanMedicine, WHO-ICTRP) were also searched (see search string for MEDLINE in the extended data). In addition, reference lists of key relevant reviews known to the review team were manually checked. The original searches were conducted in October 2024, and were updated in January 2025, April 2025, July 2025, and October 2025. Records from databases were imported into EPPI-Reviewer ( Thomas *et al.*, 2023) and deduplicated. Update searches will be performed every three months, as specified in our protocol (see Kennett *et al.*, 2025).

### Study selection

All record screening was done in EPPI-Reviewer ( Thomas *et al.*, 2023). The titles and abstracts of the identified records were examined independently by two members of the review team (JK, CF, CM, JP). Disagreements between reviewers were resolved through discussion or in consultation with a third reviewer. The full texts of all records deemed eligible were then retrieved and each screened independently by two members of the review team (JK, CF, CM, JP). Conflicts were resolved in the same way as at title and abstract stage.

### Data extraction

Data was extracted from primary studies using EPPI-Reviewer ( Thomas *et al.*, 2023). We used a piloted data extraction form and data was extracted independently by two reviewers (JK, CF, CM, SW, JP). Any differences were reconciled via discussion or through the involvement of a third reviewer.

For continuous outcomes, data was extracted as means and standard deviations at baseline and post-intervention timepoints, if available, or as change from baseline. Where standard deviation was not reported, it was calculated manually from standard error. If standard error was also missing, it was calculated from reported test statistics, confidence intervals (CIs) or alternative reported distribution ( *e.g.* median/range). We extracted number of events reported for binary outcomes and, where studies reported percentages, we converted these to absolute numbers.

We also extracted the following pre-specified effect modifiers:

• Allegiance bias (at least one of the authors is involved in the development of the CBM intervention that is tested)
• Delivery mode of CBM (in person/online/via mobile)
• Number of CBM sessions (either single or multiple)
• Cognitive bias targeted

Where authors in the included studies reported mediators of the effects of CBM, we extracted the mediator, the relevant outcome, the measure used to examine the mediating effect. We extracted raw text from the included studies on any factor reported to be a mediator.

### Risk of bias

We used the Risk of Bias 2 tool to assess each all studies for each outcome in our analysis ( Sterne *et al.*, 2019). Risk of bias assessment for each study was conducted by two independent reviewers (JK, CF, CM) and discrepancies reconciled via discussion or involvement of a third reviewer. The overall risk of bias was graded as: low risk, if no domains were rated high risk and no more than one domain was rated some concerns; high risk, if any domain was rated high risk; and some concerns, if more than one domain was rated as having some concerns and no domains were rated high risk. We used the Risk of Bias due to Missing Evidence in Network meta-analysis (ROB-MEN) tool to assess the risk of bias due to missing evidence for our primary outcome ( Chiocchia *et al.*, 2021).

### Classification of CBM interventions and control conditions

The classification of CBM interventions and control conditions was decided at an adjudication meeting held after data extraction was completed of topic experts and methodologists (TF, PC, SB, VC) who were blind to the extracted data. The lead author classified interventions and control conditions based on the information provided in the study texts and our pre-specified classifications of expected interventions in our protocol ( Kennett *et al.*, 2025). These were distributed to the expert team prior to the meeting. The adjudication panel used the intervention descriptions extracted from the included studies (including the authors’ own description of the respective interventions) and considered their intended targets and procedures to classify CBM subtypes. The panel then discussed uncertainties in the classifications of subtypes, and any disagreements were resolved via discussion.

### Data analysis

***Prioritisation of effect measures***

Where studies reported more than one measure of the same outcome we followed a predefined hierarchy for selecting the measure, prioritising in order a) the primary outcome reported by the study, b) the measure which is not self-reported, c) the measure with the best psychometric properties, and d) in the case that the measures have similar psychometric properties, the one most commonly used by studies in our review. A list of all measures reported by the included studies and our rationale for prioritising each effect measure in the analysis can be found in the extended data.

***Effect sizes***

Effect sizes were calculated as standardised mean differences (SMDs) for continuous outcomes and as odds ratios (ORs) for dichotomous outcomes. 95% confidence intervals around the pooled effect sizes were calculated for each meta-analysis.

***Data synthesis approach***

**Pairwise meta-analysis**

Firstly, we performed a quantitative synthesis via a pairwise meta-analysis using a frequentist random effects model for all CBM interventions versus control conditions. Where more than five studies contributed to the meta-analysis, we calculated the 95% confidence intervals using the Hartung-Knapp method. Restricted Maximum Likelihood (REML) was used to estimate the between study variance, τ ^2^, which was reported using 95% prediction intervals (PI).

**Network meta-analysis**

As there was significant heterogeneity between the CBM interventions and control conditions, when feasible, we conducted a network meta-analysis (NMA) using a random-effects model ( Mavridis *et al.*, 2015). We checked whether clinical and methodological characteristics that could act as effect modifiers (see investigation of heterogeneity below) were distributed similarly across comparisons to evaluate the plausibility of the assumption of transitivity.

We assumed a common heterogeneity parameter (τ ^2^) across treatment comparisons, and we assessed inconsistency using both local and global methods ( Salanti, 2012), using the SIDE (separating-indirect-evidence-from-direct-evidence) test and the design-by-treatment interaction test ( Dias *et al.*, 2010; Higgins *et al.*, 2012) respectively. Relative treatment effects were presented in forest plots (effects compared to control CBM-A) and league tables ordered by ranking based on the surface under the cumulative ranking curve (SUCRA) ( Salanti *et al.*, 2011).

**Exploration of heterogeneity**

We conducted subgroup analysis, where feasible, with our pre-specified effect modifiers: allegiance bias (at least one of the primary study authors was involved in the CBM intervention assessed in the trial); mode of delivery of the intervention; whether the intervention was single- or multi-session. We initially specified target bias as an effect modifier. However, in the final dataset there was no within group variation as CBM-A exclusively targets attentional bias and CBM-I exclusively targets interpretation bias; this subgrouping by treatment class would not have provided additional information and so the planned subgroup analysis was not conducted.

**Sensitivity analysis**

We conducted sensitivity analyses for our primary outcome by: 1) excluding studies with a high risk of bias and 2) restricting the analysis to studies that recruited participants with a diagnosis of social anxiety disorder determined by a clinical interview.

**Summary of the evidence tables**

In the protocol we stated that we would evaluate the summary of evidence at the pairwise level. Due to the heterogeneity in subtypes of CBM and to present the most comprehensive overview of the available evidence, we chose to present it at the network level. We assessed and presented within-study biases, across-study biases, indirectness, and other biases in predefined summary of evidence tables as stated in our protocol ( Kennett *et al.*, 2025). The Confidence In Network Meta-Analysis (CINeMA) framework was used to assess our confidence in the evidence ( Nikolakopoulou *et al.*, 2020). For assessing imprecision, heterogeneity, and incoherence a SMD of 0.24 was set as a conservative clinically important size of effect. This was determined based on the minimum clinically important difference suggested for improvements in general anxiety on standardised scales ( Kroenke *et al.*, 2019; Toussaint *et al.*, 2020) and for psychological interventions in other mental health conditions ( Cuijpers *et al.*, 2014).

**Synthesis of mediators**

The information on mediators was summarised descriptively. In future iterations of this LSR we may seek to examine mediation using quantitative synthesis.

**Co-production methods**

This LSR was conducted in collaboration with people with lived experience. The question was selected by members of GALENOS’ Global Lived Experience Advisory Board (GLEAB) during a prioritisation exercise, in which several questions were ranked against a criterion that was decided upon by the group. Two members of the GLEAB (RM, KO) are part of the author team for this review and contributed to the formulation of the research aims, selection of outcomes of importance, interpretation of results, and the drafting of the manuscript and plain language summary.

## Findings

### Description of included studies

The detailed report of the results in the extended data includes detailed reports of all analyses and the summary of evidence table.

Following the removal of 11,231 duplicates, 12,238 records were screened at title and abstract, of which 11,844 records were considered ineligible. 394 reports were sought for retrieval for full-text screening (see Figure 1). We could not retrieve 12 reports, leaving 382 reports to be screened at full text. From these, 256 reports were excluded for the following reasons: ineligible study design (n = 18), wrong population (n = 142), and wrong intervention (n = 50) or comparator (n = 29) (see the extended data lists of excluded studies with justifications). The full texts deemed eligible and included in our review (n = 126) include protocols, main trial papers and further publications, such as secondary analyses, of 82 eligible studies. Characteristics of the eligible studies can be found in the detailed report of the results in the extended data. 48 studies contributed data to at least one outcome in the clinical source of evidence while seven contributed to the non-clinical source of evidence.

**Figure 1.**
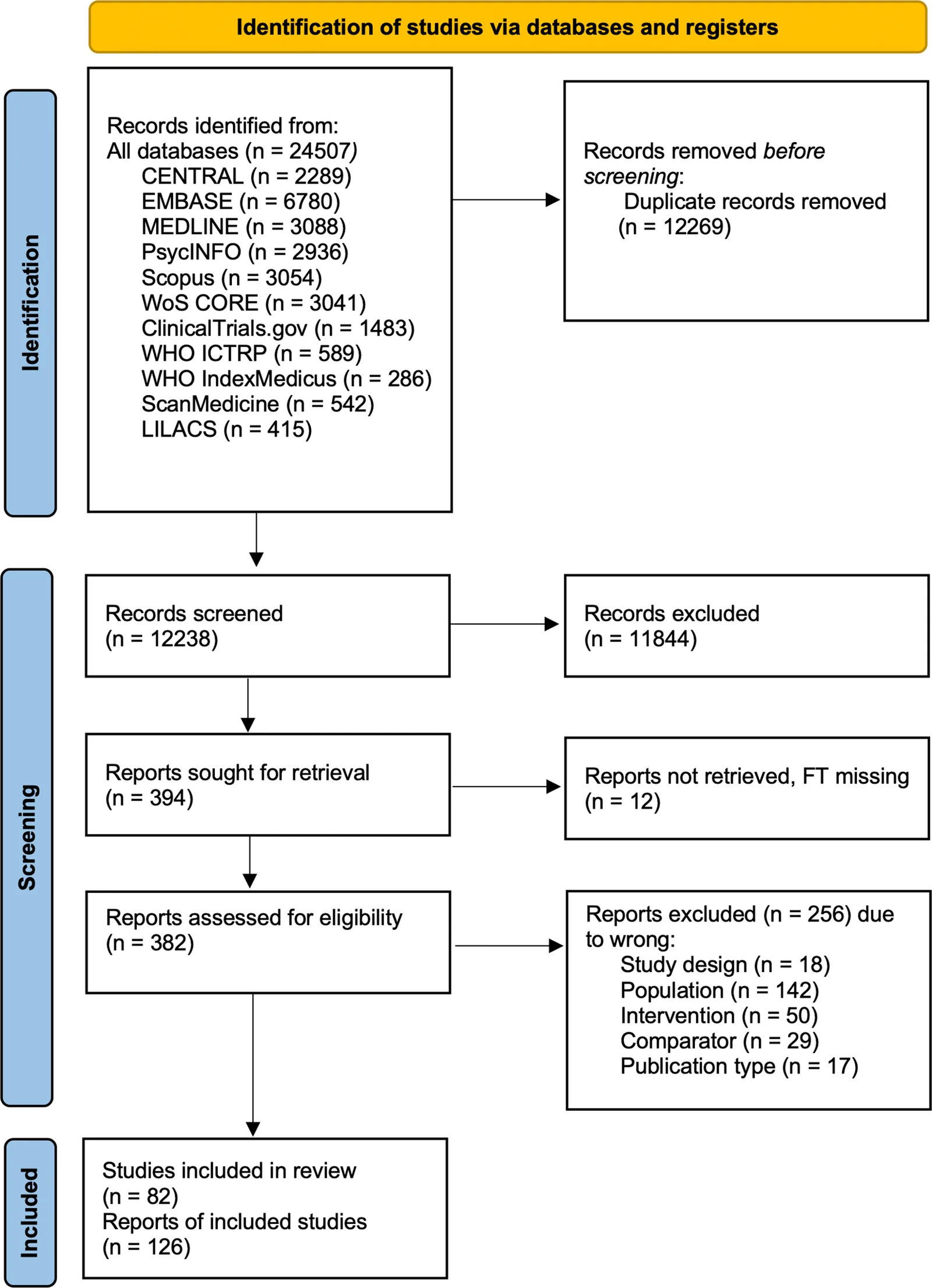
PRISMA (2020) flow diagram of study selection.

Data from these studies accounted for a total of 4,247 participants (3,373 participants in the clinical source of evidence and 874 in the non-clinical source of evidence). The mean age of participants in the clinical source of evidence was 23.4 years with 69.6% of these reported as being female. The mean age of participants in the non-clinical source was 22.9 years with 46.1% of these being reported as female. The CBM interventions included were Attention Avoidance Training (AAT); control AAT; CBM-A Cognitive Bias Modification for Attention (CBM-A); Cognitive Bias Modification for Interpretations (CBM-I); Control CBM-A; Control CBM-I; combined control; multicomponent CBM; opposite CBM-A; opposite CBM-I. Control groups included Treatment as Usual (TAU); and waitlist.

The adjudication panel was able to unanimously classify each intervention and control condition after deliberation aside from Heeren *et al.* (2011) and Ferrari *et al.* (2018). In the case of Heeren *et al.* (2011), the study consisted of four arms; a standard CBM-A arm, a control CBM-A arm, and two arms which replicated different parts of the CBM-A intervention. This dismantling of the standard CBM-A procedure created two interventions which were neither fully CBM nor fully control. As such, it was decided to not include these arms in the analysis and to analyse only the standard CBM-A and CBM-A control arms in the study. Similarly, Ferrari *et al.* (2018) used a standard AAT procedure, a control AAT procedure, and two arms which replicated different parts of the AAT intervention in an attempt to dismantle the intervention and test the effects of its individual mechanisms. These arms were also not analysed. Each intervention in the review and their classification decided by the adjudication panel can be found in the extended data. Two studies examining the efficacy of Approach-Avoidance Training (AAT) and its respective control condition were excluded from the network meta-analysis due to network disconnection.

### Clinical source of evidence

***Primary outcome – reduction in symptoms of SAD***

Pairwise meta-analysis of CBM interventions versus any control in the clinical population showed a small effect favouring CBM (SMD = −0.16, 95% CI: −0.32 to 0.00, 95% PI: −0.77 to 0.45). See the detailed report of the results in the extended data for more details. As there was a high amount of heterogeneity in the interventions and controls used in the included studies, network meta-analysis was conducted to establish the relative effects of CBM subtypes and individual control conditions. The network meta-analysis included 39 studies (52 pairwise direct comparisons) evaluating SAD symptoms in 10 distinct interventions. The network comprised various subtypes of cognitive bias modification (CBM) and comparators as (see network geometry in Figure 2). Control CBM-A is structurally similar to other forms of CBM (in terms of intervention duration, use of training tasks, and delivery) but does not train an active bias; this treatment was also well-connected with other treatments through direct evidence. As such, control CBM-A was selected as the reference treatment in our forest plot ( Figure 3).

**Figure 2.**
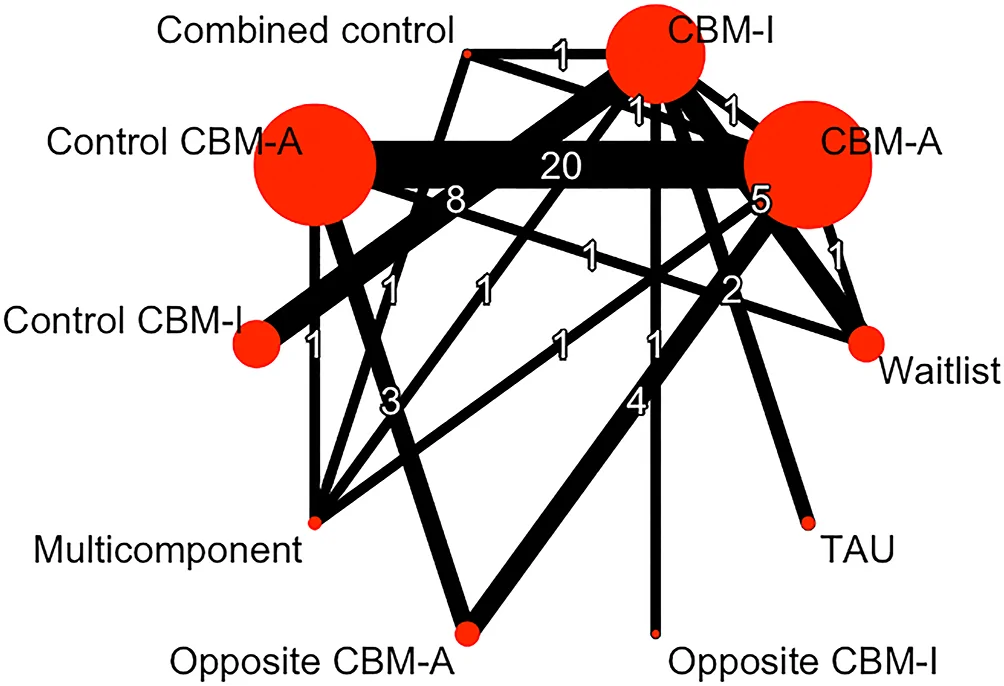
Network plot of CBM interventions for SAD. The size of the nodes is directly proportional to the number of patients, while the thickness of the lines connecting the nodes is weighted by the number of trials directly assessing the comparison. CBM-I = Cognitive Bias Modification for Interpretations; CBM-A = Cognitive Bias Modification for Attention; TAU = Treatment As Usual; Opposite CBM-I = Opposite Cognitive Bias Modification for Interpretations; Opposite CBM-A = Opposite Cognitive Bias Modification for Attention; Multicomponent = Multicomponent CBM; Control CBM-I; Control CBM-I = Control Cognitive Bias Modification for Interpretations; Control CBM-A = Control Cognitive Bias Modification for Attention.

**Figure 3.**
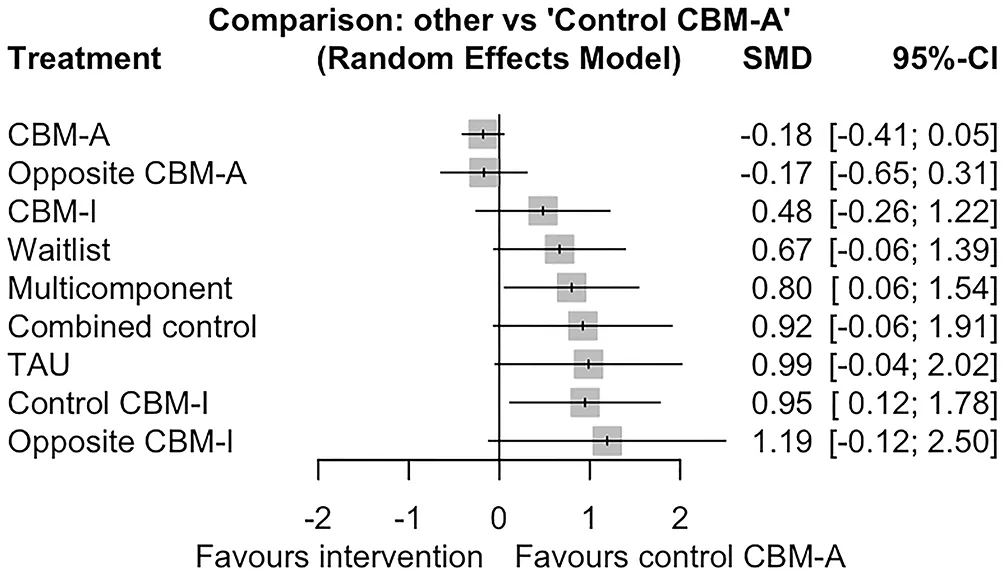
Forest plot of relative efficacy of CBM interventions for SAD (clinical source of evidence). Compared to control CBM-A. Treatments are all subtypes of CBM included in our review. Positive values represent a treatment being less effective than control CBM-A, and negative values represent a treatment being more effective than control CBM-A. SMD = standardised mean difference, CI = 95% confidence interval. Effect sizes and confidence intervals were calculated using the random effects model.

We assessed that the assumption of transitivity was plausible by inspecting relevant study characteristics and examining the distribution of the effect modifiers across the treatment comparisons (see extended data for more details). We did not detect major differences in the distribution of our pre-specified effect modifiers across comparisons. Only two of the 34 studies in the network used single session protocols and only three did not deliver the CBM intervention via computer (see data for table of study characteristics. Similarly, we only judged one study in the network to have potential allegiance bias.

Although CBM-A showed a small effect over control CBM-A (SMD = −0.18, 95% CI: −0.41 to 0.05, 95%; PI -0.76 to 1.12), it did not differentiate itself from opposite CBM-A in terms of efficacy compared to control CBM-A (SMD = −0.17, 95% CI: −0.65 to 0.31, 95%; PI: −1.02 to 1.04) ( Figure 3). Table 3 shows the relative treatment effects for all possible pairwise comparisons.

**Table 3.** Relative treatment effects for all possible pairwise comparisons estimated in the NMA for SAD outcomes (clinical source of evidence).

	CBM-A	Opposite CBM-A	Control CBM-A	CBM-I	Waitlist	Multi- component	Combined control	Control CBM-I	TAU
** Opposite CBM-A **	−0.01 CI: [−0.47; 0.45] PI: [−1.02; 1.04]								
**Control CBM-A **	−0.18 CI: [−0.41; 0.05] PI: [−0.76; 1.12]	−0.17 CI: [−0.65; 0.31] PI: [−0.87; 1.21]							
**CBM-I **	−0.66 CI: [−1.39; 0.07] PI: [−0.52; 1.85]	−0.65 CI: [−1.51; 0.20] PI: [−0.62; 1.93]	−0.48 CI: [−1.22; 0.26] PI: [−0.71; 1.67]						
**Waitlist**	−0.85 CI: [−1.57; −0.12] PI: [−0.33; 2.02]	−0.84 CI: [−1.68; 0.01] PI: [−0.43; 2.10]	−0.67 CI: [−1.39; 0.06] PI: [−0.52; 1.85]	−0.18 CI: [−0.64; 0.27] PI: [−0.84; 1.21]					
**Multi- component**	−0.98 CI: [−1.73; −0.23] PI: [−0.22; 2.18]	−0.97 CI: [−1.84; −0.10] PI: [−0.31; 2.25]	−0.80 CI: [−1.54; −0.06] PI: [−0.39; 1.99]	−0.32 CI: [−1.19; 0.55] PI: [−0.96; 1.60]	−0.13 CI: [−1.03; 0.76] PI: [−1.17; 1.44]				
**Combined control**	−1.10 CI: [−2.08; −0.12] PI: [−0.26; 2.47]	−1.09 CI: [−2.17; −0.02] PI: [−0.35; 2.53]	−0.92 CI: [−1.91; 0.06] PI: [−0.45; 2.29]	−0.44 CI: [−1.44; 0.56] PI: [−0.94; 1.82]	−0.26 CI: [−1.30; 0.79] PI: [−1.16; 1.67]	−0.12 CI: [−1.12; 0.87] PI: [−1.25; 1.50]			
**Control CBM-I **	−1.13 CI: [−1.95; −0.30] PI: [−0.12; 2.38]	−1.12 CI: [−2.05; −0.18] PI: [−0.21; 2.45]	−0.95 CI: [−1.78; −0.12] PI: [−0.31; 2.20]	−0.47 CI: [−0.84; −0.09] PI: [−0.53; 1.46]	−0.28 CI: [−0.87; 0.31] PI: [−0.82; 1.38]	−0.15 CI: [−1.10; 0.80] PI: [−1.19; 1.49]	−0.02 CI: [−1.10; 1.05] PI: [−1.41; 1.46]		
**TAU**	−1.17 CI: [−2.19; −0.14] PI: [−0.23; 2.57]	−1.16 CI: [−2.28; −0.04] PI: [−0.32; 2.63]	−0.99 CI: [−2.02; 0.04] PI: [−0.42; 2.39]	−0.50 CI: [−1.22; 0.22] PI: [−0.67; 1.68]	−0.32 CI: [−1.17; 0.53] PI: [−0.95; 1.59]	−0.19 CI: [−1.32; 0.94] PI: [−1.30; 1.67]	−0.06 CI: [−1.30; 1.17] PI: [−1.51; 1.63]	−0.04 CI: [−0.85; 0.77] PI: [−1.20; 1.28]	
**Opposite CBM-I **	−1.37 CI: [−2.68; −0.07] PI: [−0.26; 3.00]	−1.36 CI: [−2.74; 0.02] PI: [−0.33; 3.06]	−1.19 CI: [−2.50; 0.12] PI: [−0.44; 2.83]	−0.71 CI: [−1.79; 0.37] PI: [−0.73; 2.15]	−0.53 CI: [−1.70; 0.65] PI: [−0.99; 2.04]	−0.39 CI: [−1.78; 0.99] PI: [−1.31; 2.09]	−0.27 CI: [−1.74; 1.20] PI: [−1.51; 2.05]	−0.25 CI: [−1.39; 0.90] PI: [−1.25; 1.74]	−0.21 CI: [−1.50; 1.09] PI: [−1.42; 1.83]

CI = 95% confidence interval; PI = 95% prediction interval. Red indicates very low confidence in the evidence, orange indicates low confidence, yellow indicates moderate confidence according to the CINeMA assessment.

All subtypes of CBM focusing on interpretation were less effective, on average, than control CBM-A. CBM-I was found to be moderately more effective than its structurally matched control, control CBM-I, (SMD = −0.47, 95% CI: −0.84 to −0.09, 95%; PI: −0.52 to 1.46). It was also found to be more effective than opposite CBM-I, however this effect was very imprecise (SMD = −0.71, 95% CI: −1.79 to 0.37, 95%; PI: −0.73 to 2.15).

In terms of relative efficacy between the two main subtypes of CBM, CBM-I showed a moderate but imprecise effect compared to CBM-A (SMD = −0.66, 95% CI: −1.39 to 0.07, 95%; PI: −0.52 to 1.85).

There was some statistical inconsistency in the data. There were in total 13 different treatment comparisons included in loops in the network, and there was inconsistency (according to SIDE p-value <0.10) in 7, equal to 54%. The p-value from the design-by-treatment test to evaluate global inconsistency was 0, showing evidence of global inconsistency.

***Risk of bias***

Most of the studies included in our review had a moderate risk of bias. For our primary outcome, reduction in social anxiety scores, 56% of studies had a moderate overall risk of bias while 29% were designated as having a high risk of bias (see risk of bias plots in the detailed report of the results in the extended data). A key concern across outcomes was the potential bias due to selection of the reported result as very few studies had pre-specified analysis plans.

***Risk of bias due to missing evidence***

Using the ROB-MEN tool, we rated nine comparisons in our social anxiety network as having some concerns for risk of bias due to missing evidence: CBM-A versus CBM-I; CBM-A versus Control CBM-A; CBM-A versus Multicomponent; CBM-A versus Opposite CBM-A; CBM-I versus Control CBM-I; CBM-I versus Opposite CBM-I; Control CBM-A versus Multicomponent; Control CBM-A versus Opposite CBM-A; Opposite CBM-A versus Waitlist. One comparison was rated as high risk: multicomponent versus opposite CBM-A. The remainder was rated as low risk, see judgements for all comparisons and domains of the ROB-MEN tool in the detailed report of the results in the extended data.

***Subgroup analysis***

As only one study used a virtual reality headset, three used a smartphone, two used any device with internet access, two used sentences read aloud by the experimenter and the remainder used a computer, subgroup analysis by mode of delivery was not conducted. Similarly, only two studies did not use multi-session protocols and only one with suspected allegiance bias and so subgroup analysis was not conducted.

***Sensitivity analysis***

We conducted our pre-specified sensitivity analyses on our primary outcome - symptoms of SAD – for the clinical population. We restricted the analysis to only include studies with low or moderate risk of bias and to studies where individuals were diagnosed with SAD (as opposed to just having above-threshold scores on a standardised measure) it did not substantially change our results (see section 2.3.1 in the detailed report of the results in the extended data).

### Confidence in the evidence

We rated three comparisons (CBM-A versus control CBM-A, CBM-A versus opposite CBM-A, and control CBM-A versus waitlist) to have moderate confidence, 15 were judged to have low confidence, and 27 were judged to have very low confidence. This was primarily due to concerns with imprecision. All CINeMA ratings and reasons for downgrading, can be found in section 5 of the detailed report of the results in the extended data.

***Secondary outcomes***

**Depression**

Pairwise meta-analysis of CBM interventions versus control conditions for symptoms of depression in the clinical population included 15 studies (see section 2.3.2 of the detailed report of the results in the extended data) and showed no effect (SMD = 0.03, 95% CI: −0.14 to 0.19, 95% PI: −0.33 to 0.38). We conducted a network meta-analysis for symptoms of depression, with 21 studies (29 pairwise comparisons) evaluating 8 interventions. The CBM subtypes showed SMD ranging from 0.04 (95% CI: −0.14 to 0.22, 95% PI: −0.41 to 0.49) for CBM-A to 0.55 (95% CI: −0.29 to 1.40, 95% PI: −0.44 to 1.54) for control CBM-I compared to control CBM-A (see section 2.3.3 in the detailed report of the results in the extended data). There was little evidence of inconsistency from the tests for inconsistency with no inconsistent loops.

**General anxiety**

Using pairwise meta-analysis to examine 16 studies we found no effect on general anxiety (SMD = −0.01, 95% CI: −0.19 to 0.18, 955 PI: −0.48 to 0.47) and substantial heterogeneity. The included studies formed three disconnected subnetworks. See the section 2.3.3 of the detailed report of the results in the extended data for details.

**Change in bias**

Our pairwise meta-analysis of change in bias showed a moderate effect for CBM subtypes reducing bias towards negative information (SMD = −0.43, 95% CI: −0.77 to −0.09, 95% PI: −1.84 to 0.98). We conducted a network meta-analysis with 27 studies (43 pairwise comparisons) and 9 interventions (see section 2.3.4 of detailed report of the results in extended data). Compared to control CBM-A, no intervention showed a relative treatment effect excluding the null effect. The CBM subtypes had SMD ranging from −0.33 (SMD, 95% CI: −0.70 to 0.05, 95% PI: −1.47 to 0.92) in CBM-A to 0.8 (SMD, 95% CI: −0.14 to 1.74, 95% PI: −0.65 to 2.26) in Control CBM-I compared to control CBM-A. However, CBM-I was more effective than its structurally matched control, control CBM-I (SMD = −0.72, 95% CI: −1.14 to −0.29, 95% PI: −1.88 to 0.45). There was no evidence of local or global inconsistency.

**Quality of life**

Three studies contributed to our pairwise meta-analysis of quality of life scores, showing a moderate but imprecise effect favouring control (SMD = 0.46, 95% CI -0.02 to 0.94). We conducted an NMA for quality of life scores based on seven studies (14 comparisons) and seven treatments. All interventions showed similar imprecise effect sizes compared to control CBM-A, ranging from −0.51 (SMD, 95% CI -2.43 to 1.41, 95% PI: −3.67 to 2.66) for control CBM-I to 0.38 (SMD, 95% CI -0.53 to 1.29, 95% PI: −0.53 to 1.29) for opposite CBM-A (see section 2.3.4 of the detailed report of the results in the extended data).

**Acceptability (all cause discontinuation)**

Pairwise meta-analysis of CBM interventions versus control conditions for acceptability in the clinical population included 18 studies. The analysis showed a small effect favouring control (OR = 1.16, 95% CI from 0.77 to 1.63) not excluding the null effect. We conducted an NMA based on 27 studies (36 comparisons) and nine interventions. The relative treatment effects compared to control CBM-A ranged from 0.34 (OR, 95% CI: 0.03 to 3.69) for TAU to 1.65 (OR, 95% CI: 0.33 to 8.22) for combined control (see section 2.3.6 of the detailed report of the results in the extended data). While we found no evidence on inconsistency, the network consisted of a number of studies with only very few events and so interpreting these findings warrants caution.

**Tolerability**

Only two studies in the clinical source ( Ferrari *et al.*, 2018; Li & Liang, 2025) reported on dropouts due to adverse events and stated that that no participants dropped out. No studies reported details of adverse events.

**Adverse events**

One study ( Ferrari *et al.*, 2018) reported adverse events. The authors reported that in two of the CBM conditions (avoid negative training and approach positive training) participants experienced an increase in state anxiety immediately after the training phase, although not how many participants.

### Non-clinical sources of evidence

***Social anxiety***

Only two studies contributed data on SAD scores in the non-clinical source ( Enock *et al.*, 2014 using CBM-A and control CBM-A and de Voogd *et al.*, 2018 using CBM-I and control CBM-I). Pairwise meta-analysis showed a small effect not excluding the null effect (SMD = 0.09, 95% CI -0.10 to 0.29, see section 2.4.1 of the detailed report of the results in the extended data for details).

***Depression***

Only two studies reported data on depression ( de Voogd *et al.*, 2018; Vassilopoulos *et al.*, 2014). However, only one ( de Voogd *et al.*, 2018) compared CBM against control as Vassilopoulos *et al.* (2014) tested two active interventions against one another, CBM-I and opposite CBM-I. As such, pairwise meta-analysis was not conducted.

***General anxiety***

Pairwise meta-analysis of CBM interventions versus control conditions for general anxiety in the non-clinical population included two studies. The analysis showed a large effect favouring CBM (SMD = −0.76, 95% CI: −2.49 to 0.87). However, given the sparsity of data contributing to the analysis, these findings are not very informative. As only three studies contained eligible data, NMA was not conducted.

***Change in bias***

Pairwise meta-analysis of CBM interventions versus control conditions for general anxiety in the non-clinical population included four studies. The analysis showed a small effect favouring CBM (SMD = −0.23, 95% CI: −0.48 to −0.03). As only four studies contributed data, NMA was not conducted.

***Quality of life***

No studies in the non-clinical source of evidence reported on quality of life.

***Acceptability (all cause discontinuation)***

Pairwise meta-analysis of CBM interventions versus control conditions for acceptability in the non-clinical population included four studies. The analysis showed a negligible effect favouring control (OR = 0.96, 95% CI from 0.60 to 1.54). As only four studies contributed data, NMA was not conducted.

***Mediators***

Eight studies reported mediators of the effects of CBM. It was found that change in attentional bias and change in interpretive bias for CBM-A and CBM-I trials respectively mediated improvements in SAD scores during CBM training. One study ( Liu *et al.*, 2017) examined whether changes in neural activations mediated the effects of CBM-A training on SAD. They report that change in activity in the ventrolateral pre-frontal context was a significant mediator, suggesting a potential underlying neural mechanism of cognitive bias change.

## Discussion

This LSR and NMA provides a synthesis of CBM interventions for SAD with data from 39 trials contributing across ten subtypes of CBM. This is to date the first systematic review to examine the relative efficacy of subtypes of CBM for SAD using NMA and to include different subtypes of sham CBM.

We found that while CBM-A had the largest effect of reducing SAD compared to control CBM-A, it was not more effective than opposite CBM-A, the control condition designed to induce a negative bias that standard CBM-A aims to reduce. This finding questions traditional assumptions of top-down attentional bias in SAD ( Peschard & Philippot, 2016). We found that while theoretical models suggest that training attention away from negative stimuli should reduce symptoms of SAD, treatment effects of CBM-A in our NMA did not vary when participants’ attention was trained towards either neutral or positive stimuli (the intended mechanism of the intervention). Although the intervention and its control conditions did all reduce symptoms of SAD, it had little relative efficacy over control CBM-A, an condition where the procedure is matched to CBM-A, but no bias is changed. The unexpected lack of relative efficacy potentially questions the presumed mechanism behind CBM-A. It may be that mere engagement with an attentional task in the context of disorder-relevant stimuli is responsible for reductions in symptoms of SAD observed, rather than exactly where attention is directed. This echoes the findings of Lazarov *et al.* (2019) who found that attention control training (that does not seek to change a bias) outperformed attentional training that sought to actively reduce bias in individuals with PTSD. This potential for mismatch between original theoretical predictions of the mechanism of CBM-A supports more research on the importance of cognitive flexibility and its role in SAD and is important to consider in future research on CBM training for the disorder ( Arlt *et al.*, 2016; Jain *et al.*, 2024).

By contrast, we found CBM-I to be effective compared to its matched control which does not seek to change a bias, control CBM-I. In addition, we found its opposite condition, opposite CBM-I, to be less effective than control CBM-I. This key finding emphasises that the mechanism behind the therapeutic effect of CBM-I may be its intended change in cognitive bias; CBM-I was shown in our NMA to have a large effect in reduction of negative interpretation bias over control CBM-I, its matched control condition.. This supports the literature that highlights the role of interpretation as a bottom-up cognitive process in the development and maintenance of SAD ( Chen *et al.*, 2019). This also aligns with Fodor *et al.* (2020) who found a small effect for CBM-I in general anxiety, however our LSR extends these findings by showing that CBM-I performs specifically well against its task-matched condition which does not attempt to change a bias and its opposite condition. However, the lack of superiority of CBM-I over control CBM-A (and opposite CBM-A) is not straightforward to interpret. Notably, no studies contributed direct evidence to the comparisons between control CBM-I, control CBM-I, and opposite CBM-I with control CBM-A.

Another key finding of our review is the diversity of the CBM protocols that have been tested to reduce SAD. While our adjudication process with content experts and methodologists allowed us to classify interventions for the purposes of the NMA, these interventions were heterogenous in the stimuli they used, the length of training sessions, and the number and frequency of the sessions (see table of characteristics in the detailed report of the results in the extended data). The lack of established protocols for CBM training has led to numerous trials using different forms of CBM for SAD and so many of these procedures have not been replicated, as it has been found in previous systematic reviews on CBM ( Cristea *et al.*, 2015).

The finding for our secondary outcome, depression, that no CBM intervention or control condition is significantly more effective than another echoes the findings of Jones and Sharpe’s (2017) systematic review. It also highlights the limitations of these subtypes of CBM to be applied more broadly across the clinical spectrum. SAD is highly comorbid with depression, and our living systematic review did not find CBM to be effective in alleviating depression in individuals with SAD ( Koyuncu *et al.*, 2019).

For symptoms of depression and quality of life and functioning, CBM training was not more effective than sham in their respective pairwise meta-analyses, nor was any subtype significantly more effective than any other subtype in the NMA. This echoes Jones and Sharpe’s (2017) umbrella review which did not find CBM to be consistently effective in reducing depressive symptoms. In addition, while the data precluded an NMA for symptoms of general anxiety, CBM was similarly not more effective than sham in the pairwise meta-analysis.

This finding, that for individuals with SAD, CBM may be ineffective at improving symptoms of highly comorbid conditions such as depression, generalised anxiety, or the functional impairment associated with SAD, suggests a potential disconnect between mechanistic change in bias and meaningful patient outcomes ( Aderka *et al.*, 2012). The lack of available data on the effects among individuals without a mental health diagnosis is noteworthy, as it may have given us more mechanistic insights into the effects of different subtypes of CBM on these differing sets of symptoms.

The finding that no trials reported adverse events of CBM training is important. While negative events related to psychological interventions are often poorly reported and complex to attribute to the treatment in question, they have been shown to occur ( Papaioannou *et al.*, 2021). Specifically to SAD, Boettcher *et al.* (2014) highlight the potential for adverse events in internet-based psychological interventions. With the lack of information on adverse events reported in the trials in our review it is not possible to systematically evaluate whether they occur in CBM training. As per our protocol ( Kennett *et al.*, 2025), we will carry out searches for new evidence every 3 months. As we find new evidence, the authors of the last iteration of the review will assess the evidence and based on its potential to substantially change the overall findings, we will incorporate it into the review. We will report all updates using the versioning system of F1000 (Wellcome Open Research), highlight the new evidence added, and specify when each update has been performed.

### Limitations

This review had some limitations. Interpreting our findings warrants caution due to the global and local inconsistency found in the network, combined with the large heterogeneity of CBM subtypes and sparsity of data. This heterogeneity makes the results of the NMA difficult to interpret and highlights that evidence in CBM literature to date does not support definitive conclusions as to the relative efficacy of specific CBM subtypes.

In addition, the sparse and inconsistent data in both the clinical and the non-clinical samples limits the generalisability of our findings. Further to this, while we kept our eligibility criteria deliberately broad and included participants of all ages, it has been argued that cognitive biases differ by developmental stage ( Abend *et al.*, 2019; Slavny, Sebastian & Pote, 2019); as such, this may have contributed to heterogeneity in our findings. It has been argued that CBM interventions may be useful in at-risk populations to prevent anxiety or for individuals with subclinical symptoms ( Beard, 2011); however, there was inadequate data in our review to systematically assess this. In addition, the potential for reporting bias as indicated in our ROB-MEN assessment represents a limitation of the field of CBM in which we found few study protocols and variability in how results were reported. This, and our assessment of low or very low confidence in the evidence for almost all comparisons for the SAD outcome, precludes us from making strong statements about the effectiveness of the different CBM subtypes. In addition, we excluded two studies using AAT and control AAT. We deemed the exclusion of these studies to therefore be methodologically appropriate, though it limits the generalisability of the present findings to interventions within the connected evidence network. The effects of AAT should be evaluated through separate pairwise meta-analysis or future trials explicitly designed to integrate it into the broader CBM intervention framework. Due to the limited amount of studies retrieved, we did not manage to carry out a triangulation of evidence, one of the key features of the GALENOS ( Smith *et al.*, 2025). The process of triangulation aims to bring together various sources of evidence while maintaining the highest standards of methodological rigour and expert input. Triangulating evidence from different types of studies (different study designs, different populations of participants) can be particularly helpful to enable closer examination of the underlying mechanisms that underpin disorders and their treatment and to inform research prioritisation, which can identify future research routes and provide new evidence to support clinical care ( Cipriani *et al.*, 2023).

### Crowdsourcing

In this LSR we explored the potential for crowdsourcing for title and abstract screening. We trialled use of the Cochrane Crowd (see Noel-Storr *et al.* (2021) for full details of the Cochrane Crowd platform) to support the identification of relevant studies from our database searches. The use of crowdsourcing to support evidence synthesis is particularly important in LSRs which are regularly updated with new studies to evaluate. The Cochrane Crowd screened all records identified in our initial search in October 2024 following completion of specific training created by the review team and the Cochrane Crowd lead. All records screened by the crowd were also screened by members of this review team. Of these 10,198 records, the crowd identified 695 records as possibly relevant and 9503 as not relevant. Of the 123 studies identified as eligible at full text stage by the author team, the crowd correctly identified 109, resulting in 89% sensitivity. The crowd achieved 96% specificity.

While the Cochrane Crowd’s accuracy in terms of sensitivity in our review was less than that reported by Noel-Storr *et al.* (2021), this is likely due to two factors: 1. our review includes trials with non-clinical participants which were more complex to identify than traditional RCTs; and 2. the training module contained only five practice records. This was not a broad enough sample of training records given the complexity of the includable population and the fact that the crowd were being asked to assess records based on both study design and PICO characteristics. At present our evaluation of the Cochrane Crowd supports the use of crowdsourcing evidence synthesis tasks with the caveat that at least one reviewer may still have to screen records at title and abstract stage. A second evaluation of Cochrane Crowd is underway, exploring use of a modified agreement algorithm and more comprehensive training materials.

## Conclusions

Our living systematic review represents the first analysis of the relative efficacy for CBM subtypes in SAD. We found that CBM is often conducted using diverse procedures and we found few replication studies, limiting our confidence in the current evidence base. We found CBM interventions to have a small effect size in reducing symptoms of SAD and that only CBM-I was superior to its task-matched control without active bias training and its and opposite conditions. We also found CBM-A improved symptoms of SAD regardless of its underlying mechanism – the direction that bias is trained in - although this finding is complicated by the heterogeneity of the included trials. This may suggest that the mechanism of CBM-A requires a more detailed examination. We also found that few studies on CBM report adverse events, and our risk of bias assessment showed that many studies in the field are poorly reported.

## Ethics and consent

No ethical approval or consent was required for this LSR.

## Abbreviations

• AAT: Approach Avoidance Training
• CBM-A: Cognitive Bias Modification for Attention
• CBM-I: Cognitive Bias Modification for Interpretations
• CI: Confidence Interval
• Control AAT: Control Approach Avoidance Training
• Control CBM-I: Control Cognitive Bias Modification for Interpretations
• Control CBM-A: Control Cognitive Bias Modification for Attention
• GALENOS: Global Alliance for Living Evidence on aNxiety depressiOn and pSychosis
• OR: Odds Ratio
• N: number of studies
• n: number of participants
• Opposite CBM-A: Opposite Cognitive Bias Modification for Attention
• Opposite CBM-I: Opposite Cognitive Bias Modification for Interpretations
• PI: Prediction Interval
• SD: Standard Deviation
• SIDE: Separate Indirect from Direct Evidence
• SMD: Standardised Mean Difference
• SUCRA: Surface Under the Cumulative Ranking curve
• REML: Restricted Maximum Likelihood
• RoB2: Risk of Bias 2.0
• ROB-MEN: Risk of Bias for Missing Evidence in Network Meta Analysis
• TAU: Treatment As Usual

## Data availability

### Underlying data

All data underlying the results are available as part of the article and no additional source data are required.

### Extended data

Open Science Framework: Cognitive bias modification for social anxiety: a living systematic review and network meta-analysis, https://doi.org/10.17605/OSF.IO/2W69Z ( Kennett & Friedrich, 2025).

This project contains the following extended data:

• PRISMA-NMA Checklist
• Search Strategy
• Summary of evidence table
• Detailed report of the results
• Details of outcomes prioritised
• Deviations from the protocol
• List of included studies
• Information extracted on mediators
• List of intervention classifications

Data are available under the terms of the Creative Commons Attribution 4.0 International license (CC-BY 4.0).

## Software availability

We used R version 4.3.1 ( R Core Team, 2023) and the following packages; dplyr ( Wickham *et al.*, 2023); ggplot2 ( Wickham, 2016); kableExtra ( Zhu, 2024); knitr ( Xie, 2025); meta ( Balduzzi *et al.*, 2019) netmeta ( Balduzzi *et al.*, 2023); purrr ( Wickham & Henry, 2025) readxl ( Wickham & Bryan, 2023); robvis ( McGuinness & Higgins, 2019); stringr ( Wickham, 2023).

All data and code used in the cleaning and analysis process is available on GitHub ( https://github.com/galenos-project/LSR6_CBM) under the terms of the Creative Commons Attribution 4.0 International license (CC-BY 4.0).

### Credit author statement

Jaycee Kennett: Conceptualisation, Methodology, Formal analysis, Data curation, Writing – Original Draft, Project Administration.

Virginia Chiocchia: Methodology, Writing – Review and Editing, Supervision.

Claire Friedrich: Conceptualisation, Methodology, Data curation, Writing – Original Draft.

Simon E. Blackwell: Conceptualisation, Methodology, Writing – Review and Editing.

Toshi Furukawa: Conceptualisation, Methodology, Writing – Review and Editing, Supervision, Funding Acquisition.

Per Carlbring: Conceptualisation, Methodology, Writing – Review and Editing.

Spyridon Siafis: Validation, Writing – Review and Editing.

Catherine Money: Data curation, Writing – Review and Editing.

Thomy Tonia: Writing – Review and Editing.

Simonne Wright: Data curation, Writing – Review and Editing.

Kelvin Opiepie: Writing – Review and Editing.

Richardson Mojica: Writing – Review and Editing.

Olufisayo Elugbadeo: Data curation, Writing – Review and Editing.

Paulina Schenk: Writing – Review and Editing.

Micaela Santilli: Writing - Review and Editing.

Susan Michie: Writing – Review and Editing.

Janna Hastings: Writing – Review and Editing.

Hossein Dehdarirad: Methodology, Data curation, Writing – Review and Editing.

Claire Stansfield: Methodology, Data curation, Writing – Review and Editing.

James Thomas: Methodology, Software, Writing – Review and Editing.

Jen Potts: Data curation, Writing – Review and Editing, Project Administration.

Georgia Salanti: Conceptualisation, Methodology, Writing – Review and Editing, Supervision, Funding Acquisition.

Andrea Cipriani: Conceptualisation, Methodology, Writing – Review and Editing, Supervision, Funding Acquisition.
