# Supplementary material for: Cognitive bias modification for social anxiety: a living systematic review and network meta-analysis
Source: Wellcome Open Res. 2026 May 25;11:154. Originally published 2026 Feb 27. [Version 2] doi: 10.12688/wellcomeopenres.25609.2 (PMC13646545; doi:10.12688/wellcomeopenres.25609.2)
Supplement: Peer review history

# Reviewer report: Reviewer response for version 2

**DOI:** 10.21956/wellcomeopenres.29444.r171281

**Published:** 2026-10-13

**Authors:** Alexander MacLellan

**Affiliations:** 1 National Institute of Advanced Industrial Science and Technology, Tsukuba, Japan

Thank you for the opportunity to review this paper. I found the research questions to be very interesting given the apparent variety of effects in this field, and the promise of a living systematic review to be valuable. I found this to be a well written, detailed and comprehensive review of the efficacy of cognitive bias modification training for social anxiety, and I believe will be instructive for researchers designing new trials in this field. Below I give several comments which they authors may want to consider in the next version of this manuscript, organised into what I consider to be major and minor considerations.

 Major comments:

- The effect sizes are of interest, but not reflected on in the discussion. How these stack up against minimal clinically important differences or established ES’s of interest (such as the one you mention by Cuijpers et al., 2014 for example). Given your research questions, especially RQ1 and RQ2, I would have expected some kind of reflection or mention on this. The overall efficacy effect size given at the start of the Results seems weak, and whether the size of clinical/practical significance seems a relevant point for discussion.
- Building on this, you state that CBM has a small effect at reducing SAD symptoms, but this seems a fragile effect, as you state where confidence intervals over the overall effect include, but don’t cross, the null. It was also interesting to see in Table 3 that CBM-I does outperform Control CBM-I, but confidence intervals include the null when compared to the waitlist group, where traditionally we’d expect to see larger effect sizes.
- I would also hope to see, perhaps in future iterations but a reference to this now might be helpful, a consideration of training session length and frequency, given these are two considerations I have frequently seen in attention control/cognitive bias modification training literature. You already have characterised single vs multisession interventions, but knowing the duration, both in time and days, and with what frequency would be a valuable addition.

Minor comments

- In Table 2 you state that studies were included if participants ‘have not been recruited based on having a mental health diagnosis’, but makes no reference of whether elevated symptoms needed to be present, which your introduction sets up. A minor point but were there cases where studies with samples presenting low/population average levels of symptoms were included?

- There were a few tense errors in Tables 1 and 2, switching between past and future tense.
- I believe a short summary of the types of interventions, or moving the initial text from section 1.7 of your detailed discussion of results (above Table 1) to the main manuscript would help the reader keep in mind what is meant by each label.
- The extended data and other supplementary files are a fantastic resource, though I notice they are hosted on the OSF which, as I’m sure the authors are aware, will soon discontinue this function, or at the least limit their ability to update the files current stored there. If the authors feel they may be updating these files going forward, it might be helpful to switch the hosting over to another service for easy maintenance without needing to change links in the manuscript.

Yours Sincerely,

 Alexander MacLellan, PhD

Are the rationale for, and objectives of, the Systematic Review clearly stated?

Yes

Is the statistical analysis and its interpretation appropriate?

Yes

If this is a Living Systematic Review, is the ‘living’ method appropriate and is the search schedule clearly defined and justified? (‘Living Systematic Review’ or a variation of this term should be included in the title.)

Yes

Are sufficient details of the methods and analysis provided to allow replication by others?

Yes

Are the conclusions drawn adequately supported by the results presented in the review?

Yes

Reviewer Expertise:

The effectiveness of executive function trainings on mental health conditions and their symptoms, specifically depression, anxiety and social anxiety; The mechanisms and maintenance factors of social withdrawal; The effects of Stoicism informed cognitive interventions

I confirm that I have read this submission and believe that I have an appropriate level of expertise to confirm that it is of an acceptable scientific standard, however I have significant reservations, as outlined above.

# Reviewer report: Reviewer response for version 2

**DOI:** 10.21956/wellcomeopenres.29444.r163244

**Published:** 2026-10-07

**Authors:** Colette Hirsch

**Affiliations:** 1 King's College London, London, UK

This is a timely and valuable review of cognitive bias modification (CBM) interventions for social anxiety disorder (SAD). The living systematic review approach is particularly welcome in a rapidly evolving field, and the review represents a substantial undertaking. The comprehensive search strategy, duplicate screening and extraction procedures, use of network meta-analysis (NMA), and consideration of different CBM subtypes are notable strengths. The manuscript also appropriately highlights the limitations of the current evidence base and the uncertainty surrounding conclusions regarding efficacy.

 I highlight some areas for consideration.

**Characterisation of the Evidence Base**

 An issue is around how the included literature is conceptualised. The eligibility criteria focus on randomised controlled trials, whereas the search strategy refers to studies with a randomised experimental design. This distinction is particularly important in the context of the CBM literature, which was originally developed to assess the causal role of cognitive biases in maintaining emotional problems, rather than intervention research which has appropriately only developed much later.

 Indeed almost all single session CBM studies and many multi-session CBM studies were designed primarily to investigate the causal role of cognitive mechanisms rather than clinical effectiveness. Although such studies frequently employ rigorous experimental methods and randomisation, they do not necessarily address the same questions as intervention trials designed to evaluate sustained symptom change. This distinction may be relevant when interpreting the overall evidence base, particularly given the inclusion of studies varying substantially in dosage, design, and intended purpose.

 Relatedly, the inclusion criteria allow studies regardless of the number of intervention sessions. While only a small number of single-session studies appear to have contributed to the final network, the manuscript would benefit from a brief discussion of how studies designed to test short-term cognitive change with short term symptom change, relate conceptually to studies intended to achieve longer-term symptom reduction.

**Population Definitions and Research Questions**

 I think that the distinction between the populations included within the review would benefit from clarification. The manuscript states that the non-clinical source of evidence includes individuals who may have elevated social anxiety symptoms, but who have not received a diagnosis of SAD. Elsewhere, it refers to individuals without a mental health diagnosis, while the eligibility criteria refer to participants who were not recruited on the basis of a mental health diagnosis. These categories are not necessarily equivalent. Participants who have not received a diagnosis may nevertheless experience clinically significant symptoms, and participants who were not recruited on the basis of diagnosis may still meet diagnostic criteria. Greater clarity regarding the distinction between diagnosed SAD, elevated social anxiety symptoms, and genuinely non-clinical populations would improve conceptual consistency throughout the manuscript.

**Outcome Definitions**

 The manuscript ultimately clarifies that the primary outcome is change in symptom severity from pre- to post-intervention. However, because Table 1 refers initially to "change in SAD severity" while later sections distinguish symptom severity from change in diagnostic status, a slightly more explicit definition within the eligibility tables may improve readability.

**Choice of Reference Comparator in the Network Meta-analysis**

 My principal methodological question relates to the assumptions underpinning the use of Control CBM-A as the reference comparator within the network meta-analysis. The manuscript explains that Control CBM-A was selected because it was well connected within the network and shared structural similarities with other CBM interventions in terms of delivery format and training procedures. This rationale is understandable and consistent with methodological guidance suggesting that well-connected nodes are often useful reference treatments within network meta-analysis. However, my concern is less about the statistical choice of reference node itself and more about the assumptions required for the resulting indirect comparisons to be meaningful.

 Methodological guidance for network meta-analysis emphasises that the validity of indirect comparisons depends on assumptions of transitivity and comparability across the network. While the choice of reference treatment is largely a matter of model parameterisation, confidence in comparative estimates depends on whether the interventions and comparators connected through the network can reasonably be considered exchangeable with respect to important effect modifiers.

 This issue appears particularly relevant when comparing CBM-I with Control CBM-A, given the substantial differences between the intervention classes. CBM-I interventions typically involve repeated engagement with relatively extended ambiguous social scenarios, sometimes presented in auditory form, alongside the active generation and reinforcement of benign interpretations through comprehension questions and response ratings. By contrast, CBM-A interventions generally involve attentional training paradigms using brief visually presented stimuli followed by reaction-time responses to visual cues. The corresponding control conditions therefore differ considerably in content, modality, cognitive demands, participant engagement, and time.

 Given this, I was not fully convinced that the assumption of structural similarity is sufficiently justified. The manuscript appropriately acknowledges substantial heterogeneity in stimuli, session length, session frequency, and procedural characteristics across studies, as well as evidence of inconsistency within the network. These limitations seem particularly relevant when interpreting comparisons that rely primarily on indirect evidence through a common reference condition.

 More broadly, I agree that the network meta-analysis represents the best available synthesis of a highly heterogeneous literature. However, the current evidence base may not yet support strong conclusions regarding the relative efficacy of specific CBM subtypes. Given the heterogeneity across interventions and control conditions, together with the inconsistency observed within the network, I have greater confidence in comparisons against task-matched control conditions than in some of the indirect comparisons generated across intervention classes.

 Overall, the use of Control CBM-A as the reference node remains the aspect of the analysis about which I am least persuaded. My concern is not primarily statistical, but rather relates to the assumptions required to interpret indirect comparisons across conceptually distinct intervention classes. While this does not detract from the value of the review as a rigorous and important synthesis of the CBM literature, it does temper my confidence in some of the relative efficacy estimates and rankings derived from the network.

**Interpretation of Risk of Bias**

 The risk-of-bias assessment is generally appropriate. However, the discussion of concerns relating to the absence of pre-specified analysis plans might benefit from additional contextualisation.

 Many studies within the CBM literature were conducted before prospective registration became routine, particularly in the experimental field rather than clinical trials, which is where CBM research began. While the resulting risk-of-bias concerns remain entirely valid, a brief acknowledgement of the historical development of the field may help contextualise these findings for readers.

**Interpretation of CBM-I Findings**

 One interesting finding is that CBM-I demonstrated superiority over its task-matched control condition and was associated with corresponding changes in interpretation bias. In my view, this deserves greater emphasis within the plain language summary and overall discussion, as it represents one of the more theoretically coherent findings within the review and aligns closely with the proposed mechanism of the intervention.

Are the rationale for, and objectives of, the Systematic Review clearly stated?

Yes

Is the statistical analysis and its interpretation appropriate?

Yes

If this is a Living Systematic Review, is the ‘living’ method appropriate and is the search schedule clearly defined and justified? (‘Living Systematic Review’ or a variation of this term should be included in the title.)

Yes

Are sufficient details of the methods and analysis provided to allow replication by others?

Yes

Are the conclusions drawn adequately supported by the results presented in the review?

Yes

Reviewer Expertise:

NA

I confirm that I have read this submission and believe that I have an appropriate level of expertise to confirm that it is of an acceptable scientific standard.

# Reviewer report: Reviewer response for version 1

**DOI:** 10.21956/wellcomeopenres.28205.r149669

**Published:** 2026-04-15

**Authors:** Eni S. Becker

**Affiliations:** 1 Radboud Universiteit, Nijmegen, The Netherlands

I read this manuscript with great interest. It addresses an important and timely question, and the attempt to synthesise a complex and heterogeneous literature through a living systematic review and network meta-analysis is ambitious and potentially very valuable. I also appreciated the substantial methodological work that has gone into the review, including duplicate screening and extraction, risk-of-bias assessment, and the effort to compare different CBM subtypes rather than treating CBM as a single, uniform intervention.

 At the same time, I think the manuscript would benefit from clarification in several areas.

**1. Theory / content**

 My main conceptual comment concerns the scope of the review. The review is broad in important ways: it includes a wide range of CBM variants, a broad set of control conditions, and both clinical and non-clinical populations. At the same time, it is narrower than it may first appear, because it focuses on stand-alone CBM and excludes CBM delivered concurrently with psychotherapy or pharmacological treatment. This choice makes methodological sense, but it should be made much clearer from the outset. Clinically, CBM is often discussed and considered as an augmentation approach, and readers may otherwise overgeneralise the conclusions. I would therefore recommend stating more explicitly, already in the introduction and ideally also in the abstract, that this review addresses stand-alone CBM only, and that this is important for interpretation of the findings.

 Related to this, I would encourage the authors to highlight more clearly from the beginning that the review is intentionally broad, but that this breadth comes at a cost in terms of heterogeneity. This is particularly relevant for the included populations. Participants of any age were eligible, and the review combines diagnosed SAD, above-threshold symptom samples, and non-clinical participants exposed to analogue social stressors. Age seems especially important here. In the literature on cognitive biases, developmental differences matter considerably, and findings in children and adolescents are often mixed. I therefore think age should be discussed more explicitly as a potential source of variability and as a limitation on generalisability.

 I also found the discussion of the attention findings interesting, especially the observation that CBM-A did not clearly outperform opposite CBM-A. This is an important and thought-provoking result. It may be useful to place this more explicitly in the context of related work in other disorders, such as PTSD (Bar Haim), where attention training findings have also not always conformed to the original theoretical predictions. This would help readers to see that these findings may reflect a broader question about mechanism rather than an isolated anomaly in the social-anxiety literature.

**2. Methods**

 Overall, the methodological work is careful and in many respects strong. The review is registered, the search is broad and repeatedly updated, study selection and extraction were conducted independently by two reviewers, and appropriate tools were used for risk of bias and confidence in the evidence. The use of both pairwise meta-analysis and NMA is also understandable given the diversity of interventions and comparators in the field.

 My main concern here is not with the conduct of the analyses, but with the evidence base available to support them. The review necessarily draws on a highly heterogeneous literature. Interventions differ in content, delivery, dosage, and likely mechanism; populations are broad; and control conditions vary considerably. Under those circumstances, the network meta-analysis becomes difficult to interpret. The manuscript does acknowledge inconsistency, imprecision, and low confidence for many comparisons, which is appropriate. Still, I think the paper should state even more clearly that the underlying literature may not yet support strong conclusions about fine-grained relative efficacy between nodes.

 Related to this, the classification of interventions and controls deserves fuller explanation in the main manuscript. I understand and appreciate that classification was adjudicated by experts blind to extracted data, but given how much hinges on these nodes in the NMA, readers would benefit from a clearer sense of the rationale for grouping somewhat diverse procedures together.

 I was also uncertain about the reporting of the search strategy. The manuscript describes the databases searched and the general approach, but I could not easily locate the actual search terms. If these are available only in the extended materials, I would recommend signposting this much more clearly. In a systematic review, and particularly in a living review, readers generally expect the search strategy to be readily accessible, at least in an appendix or supplement.

 A further point concerns the living-review format itself. Since this is presented as a living review, I would have expected a clearer update scheme. The manuscript notes the search updates and indicates that this is version 1, but I missed a clear explanation of how often updates are planned, what will trigger a new version, and how readers should orient themselves across versions. This will become increasingly important as the evidence base develops. Relatedly, clearer version numbering and guidance for readers would be helpful.

**3. Wording / minor points**

 The manuscript is generally well written, but in several places the language becomes overly technical and difficult to follow. I noticed this already in the abstract. Terms such as “structurally matched control” are not immediately transparent without explanation, and formulations such as “with CBM subtypes targeting different cognitive biases...” are rather dense. Since this paper is likely to be read by readers beyond a narrow methodological audience, I think the manuscript would benefit from simpler and more accessible phrasing in several key places, especially in the abstract, introduction, and discussion.

 I also found Figure 3 difficult to interpret. As I understand it, all treatments are presented relative to Control CBM-A, but the rationale for choosing this reference category is not sufficiently clear, nor is the figure itself easy to understand without returning to the methods section. This may be statistically defensible, but it needs better explanation in either the text or the figure legend.

 Finally, because this is a living review, presentation and orientation are especially important. Clearer explanations of versioning, updates, and the interpretation of key figures would make the paper more accessible and more useful over time.

 Overall, this is a promising and potentially important paper. Its main strengths are the relevance of the topic, the ambition of the review, and the overall care of the methodology. The main revisions I would suggest are to clarify the scope more explicitly, especially the focus on stand-alone CBM; to discuss heterogeneity, including age, more prominently; to improve the transparency and reader guidance expected of a living review; and to simplify some of the technical language and figure presentation

Are the rationale for, and objectives of, the Systematic Review clearly stated?

Yes

Is the statistical analysis and its interpretation appropriate?

Yes

If this is a Living Systematic Review, is the ‘living’ method appropriate and is the search schedule clearly defined and justified? (‘Living Systematic Review’ or a variation of this term should be included in the title.)

Partly

Are sufficient details of the methods and analysis provided to allow replication by others?

Partly

Are the conclusions drawn adequately supported by the results presented in the review?

Partly

Reviewer Expertise:

experimental psychopathology, CBM, clinical psychology

I confirm that I have read this submission and believe that I have an appropriate level of expertise to confirm that it is of an acceptable scientific standard, however I have significant reservations, as outlined above.

# Response

**Published:** 2026-05-08

**Authors:** Jaycee Kennett

**Affiliations:** University of Oxford Department of Psychiatry, Oxford, England, UK

We thank Professor Becker for her feedback and her valuable comments. We have revised the manuscript and provided details below on the changes we have made to each point.

 Reviewer:

**1. Theory / content**

 My main conceptual comment concerns the scope of the review. The review is broad in important ways: it includes a wide range of CBM variants, a broad set of control conditions, and both clinical and non-clinical populations. At the same time, it is narrower than it may first appear, because it focuses on stand-alone CBM and excludes CBM delivered concurrently with psychotherapy or pharmacological treatment. This choice makes methodological sense, but it should be made much clearer from the outset. Clinically, CBM is often discussed and considered as an augmentation approach, and readers may otherwise overgeneralise the conclusions.

 1.1 I would therefore recommend stating more explicitly, already in the introduction and ideally also in the abstract, that this review addresses stand-alone CBM only, and that this is important for interpretation of the findings.

**Our response:** Additional text highlighting this has been added to the abstract and introduction: Abstract: *“We co-produced a living systematic review of controlled studies evaluating stand-alone CBM in participants with SAD and in non-clinical participants undergoing a social stressor, seeking to triangulate these.”* Introduction: *“While CBM is often used to augment other interventions, we have chosen to include stand-alone CBM only in order to isolate the relative efficacy of these interventions for social anxiety. This review took an intentionally narrow approach and as the review progresses into living mode and is updated, we may decide to include trials in which CBM augments other psychological interventions.”*  

 Reviewer:

 Related to this, I would encourage the authors to highlight more clearly from the beginning that the review is intentionally broad, but that this breadth comes at a cost in terms of heterogeneity. This is particularly relevant for the included populations. Participants of any age were eligible, and the review combines diagnosed SAD, above-threshold symptom samples, and non-clinical participants exposed to analogue social stressors. Age seems especially important here. In the literature on cognitive biases, developmental differences matter considerably, and findings in children and adolescents are often mixed. I therefore think age should be discussed more explicitly as a potential source of variability and as a limitation on generalisability.

**Our response:** Text discussing this has been added to the limitations section: *“In addition, the sparse and inconsistent data in both the clinical and the non-clinical samples limits the generalisability of our findings.* *Further to this, while we kept our eligibility criteria deliberately broad and included participants of all ages, it has been argued that cognitive biases differ by developmental stage (Abend et al., 2019; Slavny, Sebastian & Pote, 2019); as such, this may have contributed to heterogeneity in our findings.”*  

 Reviewer:

 I also found the discussion of the attention findings interesting, especially the observation that CBM-A did not clearly outperform opposite CBM-A. This is an important and thought-provoking result. It may be useful to place this more explicitly in the context of related work in other disorders, such as PTSD (Bar Haim), where attention training findings have also not always conformed to the original theoretical predictions. This would help readers to see that these findings may reflect a broader question about mechanism rather than an isolated anomaly in the social-anxiety literature.

**Our response:** This has been added to the discussion to support reader interpretation. *“* *This echoes the findings of Lazarov et al (2018) who found that attention control training (that does not seek to change a bias) outperformed attentional training that sought to actively reduce bias in individuals with PTSD. This potential for mismatch between original theoretical predictions of the mechanism of CBM-A* *supports more research on the importance of cognitive flexibility and its role in SAD and is important to consider in future research on CBM training for the disorder (* *Arlt et al., 2016* *;* *Jain et al., 2024* *).*  

 Reviewer:

**2. Methods**

 Overall, the methodological work is careful and in many respects strong. The review is registered, the search is broad and repeatedly updated, study selection and extraction were conducted independently by two reviewers, and appropriate tools were used for risk of bias and confidence in the evidence. The use of both pairwise meta-analysis and NMA is also understandable given the diversity of interventions and comparators in the field.

 My main concern here is not with the conduct of the analyses, but with the evidence base available to support them. The review necessarily draws on a highly heterogeneous literature. Interventions differ in content, delivery, dosage, and likely mechanism; populations are broad; and control conditions vary considerably. Under those circumstances, the network meta-analysis becomes difficult to interpret. The manuscript does acknowledge inconsistency, imprecision, and low confidence for many comparisons, which is appropriate. Still, I think the paper should state even more clearly that the underlying literature may not yet support strong conclusions about fine-grained relative efficacy between nodes.

**Our response:** Text acknowledging this limitation has been added to the limitations section: *“This heterogeneity makes the results of the NMA difficult to interpret and highlights that evidence in CBM literature to date does not support definitive conclusions as to the relative efficacy of specific CBM subtypes.”*

 Reviewer:

 Related to this, the classification of interventions and controls deserves fuller explanation in the main manuscript. I understand and appreciate that classification was adjudicated by experts blind to extracted data, but given how much hinges on these nodes in the NMA, readers would benefit from a clearer sense of the rationale for grouping somewhat diverse procedures together.  

**Our response: **Further information on this process has been added to the methods section: *“The adjudication panel used the intervention descriptions extracted from the included studies (including the authors’ own description of the respective interventions) and considered their intended targets and procedures in order to classify CBM subtypes. The panel then discussed uncertainties in the classifications of subtypes, and any disagreements were resolved via discussion.*  

 Reviewer:

 I was also uncertain about the reporting of the search strategy. The manuscript describes the databases searched and the general approach, but I could not easily locate the actual search terms. If these are available only in the extended materials, I would recommend signposting this much more clearly. In a systematic review, and particularly in a living review, readers generally expect the search strategy to be readily accessible, at least in an appendix or supplement.  

**Our response:** In addition to the link to the search strategy in the methods section, a description of all files in the extended data (including the search strategy) and a link can be found at the bottom of the manuscript.  

 Reviewer:

 A further point concerns the living-review format itself. Since this is presented as a living review, I would have expected a clearer update scheme. The manuscript notes the search updates and indicates that this is version 1, but I missed a clear explanation of how often updates are planned, what will trigger a new version, and how readers should orient themselves across versions. This will become increasingly important as the evidence base develops. Relatedly, clearer version numbering and guidance for readers would be helpful. **Our response:** Additional information has been added to the methods section and the discussion section with additional signposting to our protocol: Methods: *“Update searches will be performed every three months, as specified in our protocol (see Kennett et al., 2025).”* Discussion: *“As per our protocol (Kennett et al., 2025), we will carry out searches for new evidence every 3 months. As we find new evidence, the authors of the last iteration of the review will assess the evidence and based on its potential to substantially change the overall findings, we will incorporate it into the review. We will report all updates using the versioning system of F1000 (Wellcome Open Research), highlight the new evidence added, and specify when each update has been performed.”*  

 Reviewer:

**3. Wording / minor points**

 The manuscript is generally well written, but in several places the language becomes overly technical and difficult to follow. I noticed this already in the abstract. Terms such as “structurally matched control” are not immediately transparent without explanation, and formulations such as “with CBM subtypes targeting different cognitive biases...” are rather dense. Since this paper is likely to be read by readers beyond a narrow methodological audience, I think the manuscript would benefit from simpler and more accessible phrasing in several key places, especially in the abstract, introduction, and discussion.  

**Our response:** To make the manuscript more accessible, the phrase ‘structurally matched control’ has been changed to: “ *task-matched control condition without active bias training”* and the phrase ‘CBM subtypes targeting different cognitive biases’ has been changed to: *“Subtypes of CBM aim to train different cognitive biases and using varying procedures”.* Language has been simplified or explained throughout the abstract, introduction, discussion, and conclusion to provide more clarity to the reader.  

 Reviewer:

 I also found Figure 3 difficult to interpret. As I understand it, all treatments are presented relative to Control CBM-A, but the rationale for choosing this reference category is not sufficiently clear, nor is the figure itself easy to understand without returning to the methods section. This may be statistically defensible, but it needs better explanation in either the text or the figure legend.  

**Our response: **More explanation has been added to the figure legend and in the associated text we have clarified why this was chosen as our reference node. Legend: *“Figure 3. Forest plot of relative efficacy of CBM interventions for SAD (clinical source of evidence) compared to control CBM-A. Treatments are all subtypes of CBM included in our review. Positive values represent a treatment being less effective than control CBM-A, and negative values represent a treatment being more effective than control CBM-A. SMD = standardised mean difference, CI = 95% confidence interval. Effect sizes and confidence intervals were calculated using the random effects model.”* Results section: *“Control CBM-A is structurally similar to other forms of CBM (in terms of intervention duration, use of training tasks, and delivery) but does not train an active bias; this treatment was also well-connected with other treatments through direct evidence. As such, control CBM-A was selected as the reference treatment in our forest plot (figure 3).”*  

 Reviewer:

 Finally, because this is a living review, presentation and orientation are especially important. Clearer explanations of versioning, updates, and the interpretation of key figures would make the paper more accessible and more useful over time.  

**Our response: **As detailed above, more information on the living mode of the review has been added to the discussion and methods section, including signposting to our versioning system on Wellcome Open Research.   We hope these revisions adequately address the reviewer's concerns and have substantially improved the manuscript.

# Reviewer report: Reviewer response for version 1

**DOI:** 10.21956/wellcomeopenres.28205.r149673

**Published:** 2026-03-24

**Authors:** Ian S Penton-Voak

**Affiliations:** 1 University of Bristol, Bristol, England, UK

This is a valuable living systematic review of cognitive bias modification (cbm) studies for Social Anxiety Disorder (SAD) as the primary outcome. Other outcomes include co-morbid psychiatric conditions (e.g. depression) and intermediate psychological outcomes (e.g. changes in cognitive bias as a result of the modification techniques). 

 The authors chose a network meta-analysis to compare two forms of putatively therapeutic cognitive bias modification (for attention and interpretation) and various controls & comparators (inc. TAU, sham CBM and ‘opposite’ CBM in which participants are trained in the non-therapeutic direction).

 The number of studies included, and statistical decisions made (e.g. Hartung-Knapp method; ROB-MEN) are conservative and appropriate. The selection of studies and classification of interventions involved a large and expert team of co-authors, and was clearly described.

 The results of the study are interesting and clearly reported. They show a lack of strong evidence that any form of CBM is particularly effective at reducing symptoms of SAD. Evidence for engagement with the putative therapeutic targets (ie. the cognitive biases) is better, which calls into question their causal role in anxiety disorders – especially as effects of CBM-A are similar irrespective of training direction. The network analysis is particularly useful here, although comparing across the interventions although some of these results are hard to interpret (particularly those comparing CBM-i against opposite CBM). Clearly heterogeneity of studies and risk of bias is high in this research area.

 It was interesting to see the use of the Cochrane crowd platform for potential title and abstract screening for future researchers.

 I would like to know the schedule for updates of this systematic living review.

Are the rationale for, and objectives of, the Systematic Review clearly stated?

Yes

Is the statistical analysis and its interpretation appropriate?

Yes

If this is a Living Systematic Review, is the ‘living’ method appropriate and is the search schedule clearly defined and justified? (‘Living Systematic Review’ or a variation of this term should be included in the title.)

Partly

Are sufficient details of the methods and analysis provided to allow replication by others?

Yes

Are the conclusions drawn adequately supported by the results presented in the review?

Yes

Reviewer Expertise:

psychology, psychological interventions including CBM, emotion perception.

I confirm that I have read this submission and believe that I have an appropriate level of expertise to confirm that it is of an acceptable scientific standard.
